# A deep neural network and transfer learning combined method for cross-task classification of error-related potentials

**DOI:** 10.3389/fnhum.2024.1394107

**Published:** 2024-06-12

**Authors:** Guihong Ren, Akshay Kumar, Seedahmed S. Mahmoud, Qiang Fang

**Affiliations:** Department of Biomedical Engineering, Shantou University, Shantou, China

**Keywords:** error-related potentials, transfer learning, transformer, cross-task classification, brain-computer interface

## Abstract

**Background:**

Error-related potentials (ErrPs) are electrophysiological responses that naturally occur when humans perceive wrongdoing or encounter unexpected events. It offers a distinctive means of comprehending the error-processing mechanisms within the brain. A method for detecting ErrPs with high accuracy holds significant importance for various ErrPs-based applications, such as human-in-the-loop Brain-Computer Interface (BCI) systems. Nevertheless, current methods fail to fulfill the generalization requirements for detecting such ErrPs due to the high non-stationarity of EEG signals across different tasks and the limited availability of ErrPs datasets.

**Methods:**

This study introduces a deep learning-based model that integrates convolutional layers and transformer encoders for the classification of ErrPs. Subsequently, a model training strategy, grounded in transfer learning, is proposed for the effective training of the model. The datasets utilized in this study are available for download from the publicly accessible databases.

**Results:**

In cross-task classification, an average accuracy of about 78% was achieved, exceeding the baseline. Furthermore, in the leave-one-subject-out, within-session, and cross-session classification scenarios, the proposed model outperformed the existing techniques with an average accuracy of 71.81, 78.74, and 77.01%, respectively.

**Conclusions:**

Our approach contributes to mitigating the challenge posed by limited datasets in the ErrPs field, achieving this by reducing the requirement for extensive training data for specific target tasks. This may serve as inspiration for future studies that concentrate on ErrPs and their applications.

## 1 Introduction

Error-related potentials (ErrPs) are a series of electrophysiological reactions that occur naturally in response to perceived wrongdoing or unexpected events in humans (Kumar et al., 2019). Usually, an electroencephalogram (EEG) with distinctive waveform characteristics and a temporal window can show these reactions. Milekovic et al. (2012) conducted a study on ErrPs in the context of simulated continuous brain-computer interface (BCI) control tasks. They found strong error-related neural responses in both high-frequency and low-frequency components of human surface EEG recordings. The study investigated two types of errors: (i) execution errors resulting from inaccurate decoding of the participants' movement intentions, and (ii) outcome errors due to failure to achieve the intended movement goal. The experiment involved four participants who took part in four or more experimental sessions. The findings from this study indicate that ErrPs are reliable neuro-electrophysiological signals in humans.

Currently, ErrPs are under continuous study in numerous application-oriented fields (Yasemin et al., 2023). In the field of neuroscience, ErrPs have been widely used to explore various neural processes such as cognitive control, learning, decision-making, and error detection. In cognitive control, Compton et al. (2013) investigated the hypothesis of a common system of cognitive and emotional self-regulation with data from 83 subjects. Grammer et al. (2018) collected data across 6 months from 49 children aged 4–6 years to study developmental changes in skills related to cognitive control in children. Regarding learning, Kopp and Wolff (2000) measured data from 16 university students to verify the trial-by-trial error correction mechanism specified by the error-driven learning rules of humans in a certain type of emergency judgment task. In decision-making, Hewig et al. (2007) studied the association between error-related negativity, risk-taking, and decision-making behavior using a computer blackjack gambling task with 18 university students. Perri et al. (2016) investigated the process of neural adjustment after error by performing a Go/No-go task on 108 subjects. In error detection, Spüler and Niethammer (2015) recorded data from 10 participants to study error detection in continuous cursor control tasks. In the field of psychology, ErrPs can inform the development of psychopathology and risk models, and Hajcak et al. (2019) used them for the programmatic research of error-related negativity and anxiety. Additionally, ErrPs have been extensively utilized in the development of control interfaces that rely on EEG activities (Kumar et al., 2019). Through these interfaces, individuals can manipulate external devices to perform specific tasks. For example, Cruz et al. (2017) developed a BCI speller with dual automatic error correction by combining ErrPs with P300. Kalaganis et al. (2018a) integrated ErrPs with gaze data to propose a gaze keyboard with an error detection mechanism. Ferracuti et al. (2023) developed an intelligent wheelchair with safe autonomous navigation capabilities by using error-related signals as additional inputs for wheelchair navigation algorithms. These interfaces may provide more autonomy and independence to individuals with mobility disabilities. Furthermore, in the fields of machine learning and reinforcement learning, ErrPs can be used as feedback or incentives to help intelligent systems learn and make decisions (Kim et al., 2017; Xu et al., 2021; Xavier Fidêncio et al., 2022). By identifying ErrPs, these systems can detect erroneous behaviors and make timely strategy adjustments to improve performance (Chavarriaga and Millan, 2010; Salazar-Gomez et al., 2017).

Despite the fact that research on ErrPs-based applications keeps growing, a number of challenges still need to be overcome before these applications can be fully deployed and reach their full potential. One of the primary challenges is the poor classification performance of ErrPs. EEG signals have a low signal-to-noise ratio and are highly non-stationary, making them vulnerable to a variety of factors such as environmental conditions, individual differences, session changes, and differences in EEG paradigms. This increases the difficulty of ErrPs classification, which leads to the suboptimal performance of existing ErrPs-based applications. Only by enhancing ErrPs' classification performance can their full application potential in numerous fields be realized. Nowadays, many researchers are experimenting with a variety of classical machine learning (CML) and deep learning (DL) algorithms for ErrPs classification (Gao et al., 2022).

The most popular CML algorithms are linear discriminant analysis (LDA) and support vector machines (SVM). In their work, Bhattacharyya et al. (2017) developed a decoder utilizing LDA, quadratic discriminant analysis, and logistic regression. This decoder was designed for the single-trial classification of EEG features in a cohort of 16 participants and subsequently evaluated on an additional set of 10 independent participants. The study by Kumar et al. (2021) employed a methodology that combined xDAWN spatial filtering and SVM to categorize ErrPs. Similarly, Usama et al. (2022) utilized an LDA classification approach to distinguish single-trial ErrPs elicited by stroke patients.

Although the methods based on the CML algorithms outlined above have shown some results in terms of classification performance when time windows, filters, and electrode channels are manually chosen, these approaches still have drawbacks. One major shortcoming is that when using these methods, the feature extraction and classification stages need to be performed independently. This independence implies a need for more a priori knowledge to attain more favorable outcomes (Vallabhaneni et al., 2021). Therefore, feature extraction methods that rely on manual selection may not be able to fully capture the complex details in the ErrPs signal, resulting in poor classifier performance.

DL algorithms can partially address this deficiency since they operate as data-driven end-to-end algorithms. Over the past few years, there has been a rapid and widespread development of DL algorithms, especially in fields such as computer vision (CV) and natural language processing (NLP). Simultaneously, in certain Brain-Computer Interface (BCI) paradigms like motor imagery (MI) and seizure detection, DL algorithms are emerging as potent tools. Altaheri et al. (2023) conducted a systematic investigation on the classification of MI EEG data using DL algorithms across the last decade. The results show that the DL approach can employ a multi-level neural network model to automatically learn advanced and complex latent features from the raw MI EEG data, removing the need for preprocessing and feature extraction in traditional approaches. Shoeibi et al. (2021) presented an in-depth review of research on seizure detection using various DL algorithms. They emphasized that semi-supervised and unsupervised learning methods can be used to overcome dataset size constraints during the development of seizure detection models. Owing to the increasing adoption of DL algorithms in EEG decoding tasks and their noteworthy performance, researchers have started exploring their application for classifying ErrPs signals. This exploration is motivated by the anticipation of improving decoding performance. Bellary and Conrad (2019) presented a deep CNN architecture designed for the classification of ErrPs. Following this, Parashiva and Vinod (2020) introduced an artificial neural network (ANN) classifier, bifurcated into two stages, for the detection of ErrPs from EEG data within a single trial. Subsequently, Kumar et al. (2021) proposed a double transfer learning strategy utilizing CNNs to classify ErrPs in stroke survivors. Similarly, Usama et al. (2022) employed an ANN to classify single-trial ErrPs generated by stroke patients. Lastly, Gao et al. (2022) presented a CNN architecture incorporating an attention structure for the classification of ErrPs.

Intuitively, these ErrPs detection methods based on DL outperform traditional approaches. From a practical application standpoint, there is a strong demand for a general ErrPs detection method capable of identifying ErrPs across diverse tasks and subjects. However, a significant portion of current methods is tailored for specific tasks, hindering their ability to meet the robust generalization requirements essential for a comprehensive ErrPs detection method (Yasemin et al., 2023). These limitations arise from the constraints imposed by specific datasets, the inherent individual differences in EEG signals, and the diversity of task types. In the context of the DL approaches, it is reasonable to assume that increasing the size of datasets can contribute to enhancing the robustness of the detection model and improving the accuracy of ErrPs detection. However, in comparison to other fields such as image, text, and speech processing, acquiring data for ErrPs is relatively costly. Obtaining reliable ErrPs necessitates a highly specialized laboratory setup and technical support, encompassing well-designed EEG experimental paradigms, sophisticated EEG acquisition equipment, and professional data preprocessing technologies. Moreover, the accessibility of EEG data is often constrained by participants' privacy concerns. These factors collectively pose significant challenges to acquiring large-scale datasets, thereby impeding the effective utilization of DL techniques for ErrPs detection.

To address the aforementioned issues, this paper introduces an innovative solution. The fundamental idea of the proposed solution is derived from transfer learning methods applied in the fields of CV and NLP. Transfer learning is a machine learning approach whose core idea is to transfer knowledge learned from one domain's task to another domain's task, even if these two domains have different data distributions (Pan and Yang, 2009; Zhuang et al., 2020). Specifically, the solution proposed in this study is to pre-train the proposed deep neural network model using the existing public dataset as training data, and then use only a small amount of data from the new EEG task to fine-tune the pre-trained model to accommodate the ErrPs detection of new participants in the new EEG task. Through an exhaustive literature review, this work is highly original. It is worth emphasizing that the most substantial distinction between this study and other existing ErrPs detection research is that this study endeavors to introduce a general method based on the DL model and transfer learning technique to classify ErrPs derived from various EEG tasks, while most other studies are only focused on specific EEG tasks. Our approach can somewhat alleviate the issue of limited datasets in the ErrPs area by significantly lowering the demand for training data for target EEG tasks. This may provide some inspiration for future studies based on ErrPs and their applications. The details of the proposed method will be expanded in the Methods section.

The remainder of this paper is organized as follows: Section 2 briefly describes the datasets used, the data preprocessing steps, the proposed method and DL architectures, as well as the model evaluation metrics. Section 3 presents the results obtained using the proposed method. Section 4 presents a comprehensive comparison between the proposed method and the other existing approaches and a wide discussion. Finally, the whole work is concluded in Section 5.

## 2 Materials and methods

### 2.1 Datasets

The proposed method underwent evaluation using three publicly available datasets, for which the data epochs and labels are either provided or can be extracted. These datasets were selected based on their availability as publicly accessible ErrPs datasets and their prevalence in ErrPs classification studies, allowing for reproducibility and facilitating comparisons with existing methods. Each of these datasets is accessible for download from the online databases (Chavarriaga and Millan, 2015; Kalaganis et al., 2018b; Cruz et al., 2020). These three datasets include two types of ErrPs datasets, namely the observation-ErrPs dataset and the interaction-ErrPs dataset. Among them, the observation-ErrPs dataset refers to the moving cursor dataset in the Brain/Neural Computer Interaction (BNCI) Horizon 2020 project. The interaction-ErrPs dataset refers to the Lateral Single Character (LSC) speller and Gaze speller datasets. Table 1 presents the details of the datasets, which are further elucidated below.

**Table 1:** Properties of datasets.

**Datasets**	**ErrPs type**	**Subjects (mean age)**	**EEG channels**	**Sampling rate (Hz)**	**Trial duration (s)**
BNCI moving cursor	Observation-ErrPs	6 (27.8 ± 2.23)	64	512	1
LSC speller	Interaction-ErrPs	8 (30.1)	12	256	1
Gaze speller	Interaction-ErrPs	10 (32 ± 4)	61	256	0.5

*BNCI moving cursor* (Chavarriaga and Millan, 2015): contains data from six participants [one female, mean age 27.8 ± 2.23]. In this work, the authors devised a cursor navigation paradigm that evaluates whether a similar error-related signal is generated when a human user monitors the performance of an external agent that he or she cannot control. During the experiment, each participant was asked to sit in front of a screen displaying a moving cursor and a target location and to observe the direction in which the cursor moved. If the moving cursor moved away from the target location, it would generate a series of error-related signals. Otherwise, they would generate some correct-related activities as well as other common signals. Every participant underwent two sessions of such an experiment. All data in this dataset were recorded using the Biosemi ActiveTwo system at a sampling rate of 512 Hz. According to the standard 10/20 international system, the authors used a total of 64 electrodes to finish this work. For more details of the experiment, see Chavarriaga and Millan (2010).

*LSC speller* (Cruz et al., 2020): contains data from seven able-bodied participants (S1–S6, S9) and one tetraplegic participant (P1) with medullar injury (C4/C5 level) with a mean age of 30.1. This dataset was obtained in a P300-based spelling task, which consists of two sessions and three phases. Among them, the ErrPs in the first session are used to detect whether the symbols the speller produces are consistent with the user's consciousness, and the second session's ErrPs are used to verify whether the speller's output following system correction is consistent with the user's consciousness. The system does not need to correct the errors if ErrPs are not found in session 1. Therefore, ErrPs in session 2 can only be identified by the system if ErrPs in session 1 are detected. Every participant in the dataset has data for two sessions. All data were recorded using a g.USBamp bioamplifier with 12 electrodes at a sampling rate of 256 Hz. For more details of the experiment, see Cruz et al. (2017).

*Gaze speller* (Kalaganis et al., 2018b): contains data from 10 participants [four female, mean age 32 ± 4]. This dataset is derived from a gaze-based keyboard paradigm composed of an eye-tracking system. At the beginning, participants were required to gaze at the desired letter. Once he or she completed the 500-ms continuous fixation interval, the key press was registered, and the associated visual indication appeared. The electrophysiological responses following this indication were used to detect typing errors. Unlike the previous two datasets, there is only one session of data per participant in this dataset. All data were recorded at a sampling rate of 256 Hz using the EBNeuro EEG device with 61 electrodes placed according to the standard 10/20 international system. For more details of the experiment, see Kalaganis et al. (2018a).

### 2.2 Data preprocessing

As EEG signals are non-stationary and have a low signal-to-noise ratio, data pre-processing is essential to the classification of EEG signals. Due to the difference in the number of electrode channels, sampling rate, and duration time of epochs, we preprocessed the three datasets separately to ensure that the data dimensions, i.e., the number of electrode channels × the number of sampling points, remained consistent. The specific steps are as follows.

1. First of all, as in most studies, we bandpass-filtered the BNCI moving cursor and LSC speller datasets, respectively, restricting their frequencies to 1–10 Hz using a fourth-order Butterworth filter (Chavarriaga and Millan, 2010; Cruz et al., 2017; Lopes-Dias et al., 2019). Since the Gaze speller dataset only contains preprocessed epoch data, we did not filter this dataset.
2. Then, the epoch corresponding to each event in the BNCI moving cursor and LSC speller datasets was extracted from the EEG data using MATLAB. The time window is one duration long and spans from the start of the event to its end. Each epoch corresponds to an array of 2D shapes (the number of electrode channels × the number of sampling points).
3. The next operation was downsampling. Based on the investigation of Yasemin et al. (2023), all epochs extracted from the BNCI moving cursor and LSC speller datasets were downsampled to 64 Hz, which is a common choice for classification purposes in BCI studies (Ferrez and Millán, 2008; Chavarriaga and Millan, 2010; Omedes et al., 2013; Iturrate et al., 2015; Iwane et al., 2016; Bevilacqua et al., 2019). As for the preprocessed Gaze speller dataset, which had a duration of 0.5 s per epoch, the epochs were downsampled to 128 Hz to ensure consistent dimensions across the datasets. After the downsampling operation, the dimension of epochs in the three datasets became (the number of electrode channels × 64). Although downsampling may result in some loss of underlying information, it offers two distinct advantages: first, it can reduce the data dimension, thereby reducing the amount of computation in the training process of the neural network model; and second, the data dimensions of different datasets can be unified so as to facilitate subsequent processing.
4. Then, there's channel selection. All channels and two channels are the two primary channel selection types that are most frequently utilized in ErrPs classification research. Although selecting data from all channels obtains more raw information than selecting data from two channels, it increases dimension and is vulnerable to the curse of dimensionality difficulties. Since the frontocentral medial region of the human brain has been shown to be active during error monitoring and processing, many ErrPs-related studies just employ data from channels in this region for various analyzes (Herrmann et al., 2004). This might drastically decrease duplicate data and calculations. Thus, in this study, only the data from two channels was used in the following analysis. For the BNCI moving cursor and Gaze speller datasets, the most commonly used FCz and Cz channels were selected. For the LSC speller dataset, since there was no data available for the FCz channel, the Fz channel is used in place of the FCz channel, just as it was in the study (Yasemin et al., 2023). After completing the channel selection, each epoch's dimension became (2, 64).
5. Finally, to facilitate the subsequent feature extraction, the amplitude value corresponding to each sampling point was expanded by 1,000 times.

### 2.3 Model design

#### 2.3.1 Convolutional layer

The convolutional layer is one of the indispensable parts of the CNN algorithm (Bouvrie, 2006). Its main function is to extract the features of input data by performing a convolution operation. The convolutional layer has a special characteristic named local connectivity, i.e., the convolutional kernel can only perform an operation with one part of the input feature map each time (Sakib et al., 2019). Only by increasing the number of convolutional and pooling layers can the model learn more comprehensive information (He et al., 2016).

#### 2.3.2 Pooling layer

The pooling layer can reduce overfitting by applying the nonlinear transformation to the entire network model and can lower parameters by removing some redundant information (Sakib et al., 2019). Average pooling (Liu et al., 2023) is a popular pooling approach that uses the average value of all the eigenvalues in the range of the pooling kernel as the characteristic after pooling.

#### 2.3.3 Transformer encoder

A transformer encoder includes a multi-headed self-attention layer and a feed-forward layer. Behind these layers, there are some connections that resemble residual neural networks (He et al., 2016), which are utilized to combine the input vector and the calculated output vector before being standardized in a batch. The multi-headed self-attention mechanism in the transformer encoder can instantly calculate the data for various points on each sequence and obtain a global, comprehensive representation without reference to the previous position's output. Due to the multi-headed self-attention layer's ability to run in parallel on the GPU, model training can be much more effective. The more specific details about the transformer encoder and multi-headed self-attention mechanism can be found in the literature (Vaswani et al., 2017). In recent years, transformer architecture has demonstrated advantages in handling sequential data and modeling long-distance interdependence in a variety of applications (Devlin et al., 2018; Achiam et al., 2023). This is particularly important for ErrPs detection, as the ErrPs are essentially time series. However, it is difficult to find published research on the performance of transformer-based models in ErrPs classification tasks. Given Transformer's benefits in processing sequential tasks, the Transformer-based architecture may help to improve the performance of ErrPs signal classification.

#### 2.3.4 Proposed architecture

**Figure 1:**
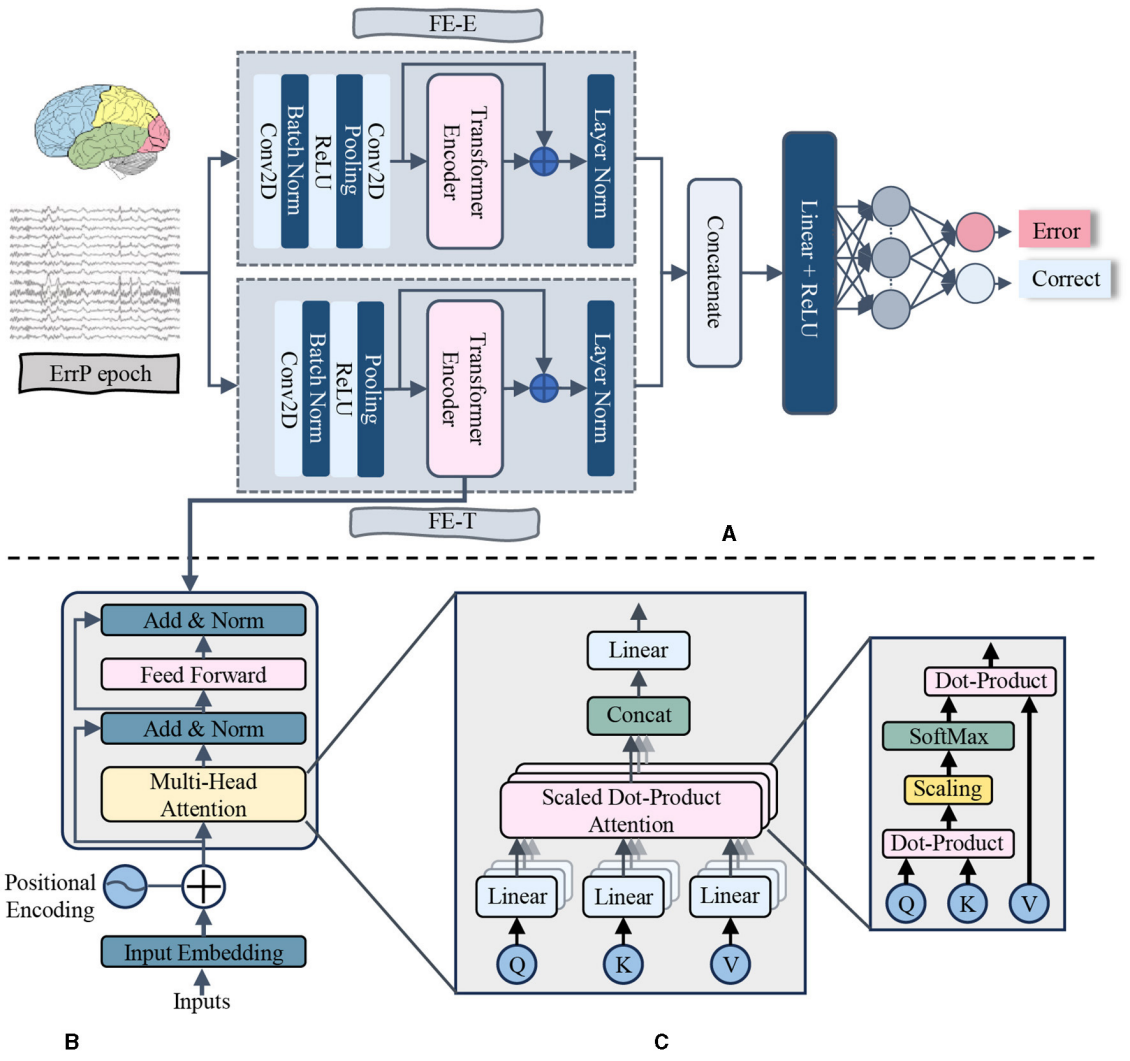
The diagram of proposed model. The data after preprocessing was input into the FE-E and FE-T module respectively with a shape of number of electrodes × number of sampling points. Then, the two feature maps produced from these two feature extraction modules were concatenated together to obtain the fusion features. Following that, a Flatten layer was used to flatten the feature matrix before inputting it into the Dense layer. Finally, the probability of each class was calculated by the SoftMax activation function to make a final decision on classification. To avoid overfitting and boost generalizability, a Batch Normalization (BN) layer was introduced after each convolutional layer in the proposed model. **(A)** Proposed model. **(B)** Transformer encoder layer. **(C)** Multi-head attention.

To classify ErrPs, we introduced a neural network model that combines convolutional layers and transformer encoder layers. The primary components of the proposed model are the Electrode Feature Extraction (FE-E) and Time Series Feature Extraction (FE-T) modules. The overall pipeline of the proposed method is illustrated in Figure 1, and the key structural parameters are provided in Table 2.

**Table 2:** The key parameters of the proposed model.

**Module**	**Layer**	**Filters**	**Kernel size**	**Padding**	**Stride**	**Head**	**Input**	**Output**
FE-E 1	Conv2d	64	(1, 33)	Valid	1	-	[B, 1, 2, 64]	[B, 64, 2, 32]
FE-E 2	AvgPool2d	-	(1, 4)	-	-	-	[B, 64, 2, 32]	[B, 64, 2, 8]
FE-E 3	Conv2d	64	(1, 8)	Valid	1	-	[B, 64, 2, 8]	[B, 64, 2, 1]
FE-E 4	TransformerEncoderLayer	-	-	-	-	4	[B, 2, 64]	[B, 2, 64]
FE-T 1	Conv2d	64	(2, 33)	Valid	1	-	[B, 1, 2, 64]	[B, 64, 1, 32]
FE-T 2	AvgPool2d	-	(1, 16)	-	-	-	[B, 64, 1, 32]	[B, 64, 1, 2]
FE-T 3	TransformerEncoderLayer	-	-	-	-	4	[B, 2, 64]	[B, 2, 64]
FC 1	Linear	64	-	-	-	-	[B, 256]	[B, 64]
FC 2	Linear	2	-	-	-	-	[B, 64]	[B, 2]

In the FE-E module, the input data was regarded as a sequence of electrodes, each of which was similar to a word vector with a feature dimension equal to the number of sampling points. Firstly, local features along the temporal dimension are captured using a convolutional layer. The average pooling layer is then applied in order to increase the receptive field. After that, a convolutional layer with 64 convolutional kernels receives the feature map that was produced. The main purpose of this convolution stage is to obtain global information regarding each electrode's temporal aspect. Each electrode feature's size is reduced to 1 after these layers. The global information obtained from the 64 convolutional kernels is used as the electrode eigenvalues, which means that each electrode possesses all of the temporal serial information. Finally, the generated feature map is fed into the transformer encoder layer, where the self-attention mechanism produces a high-level feature representation that includes the correlation between different electrode signals.

In the FE-T module, the input data was regarded as a sequence of sampling points, each of which was similar to a word vector with a feature dimension equal to the number of electrodes. The first step involves extracting complete characteristics in the electrode dimension using a convolutional layer. Average pooling is then utilized to simplify the sequence and minimize the model's computation. In order to make the dimension of the feature map learned by the FE-T module consistent with that of the FE-E module for subsequent feature fusion, the pooling operation downsamples the number of sample points to the same number of electrode channels as the input ErrPs data. Subsequently, a feature map of dimension (2, 64) is produced by using the data obtained from 64 convolutional kernels as a feature for every sampling point. In order to further extract the global features of the time dimension, the feature map is finally sent into the transformer encoder layer.

In the fields of NLP and CV, the learning capacity of neural networks increases exponentially with the depth of the network model in a certain range. However, the deeper network structure is often faced with the problems of gradient disappearance and gradient explosion (Bengio et al., 1994; Glorot and Bengio, 2010), which lead to the deterioration of information transmission ability. To avoid the difficulties mentioned above, in both the FE-E and FE-T modules, the output features of the transformer encoder layer are combined with the input features of the corresponding transformer encoder layer to obtain the final features. This design is inspired by residual connections.

After the feature extraction of the electrode dimension and the time dimension is completed, the two extracted features are concatenated to form the channel time fusion feature. Ultimately, the fusion features are flattened and fed into a classifier with two fully connected layers for classification. To prevent overfitting, we added a batch normalization layer after each convolutional layer, a layer normalization layer after the last layer of the FE-E module and the FE-T module, and a dropout layer after both fully connected layers of the classifier.

### 2.4 Proposed training strategy

A representative method in transfer learning, fine-tuning, is used to train the proposed model. Fine-tuning refers to performing additional training on a model that has been trained on a large-scale dataset to adapt to new tasks or datasets (Tajbakhsh et al., 2016). The advantage of fine-tuning lies in its capacity to leverage the common features acquired by the pre-trained model to accelerate the model's training process for novel tasks. Additionally, it can enhance the model's adaptability with only a small amount of labeled data from the target task. In this study, the BNCI moving cursor dataset was used as the dataset of the target task, and the LSC speller and Gaze speller datasets were used as the datasets of the two source tasks, respectively. Model training consists of two parts: pre-training and fine-tuning. The important settings related to training will be introduced next.

*Pre-training*: The LSC speller and Gaze speller datasets were used to pre-train the proposed model, respectively.

Table 3 displays the number of epochs extracted from each dataset. It is obvious that the number of epochs corresponding to erroneous events is much lower than the number of epochs corresponding to correct events. Numerous studies on deep learning have shown that neural network classifiers have a tendency to classify test samples into categories with more data when the data sample categories are imbalanced. A variation of the Synthetic Minority Over-sampling Technique (SMOTE), known as SVMSMOTE, was used to address this issue. In comparison to the commonly used SMOTE algorithm, the SVMSMOTE technique aims to leverage the SVM algorithm to identify the decision boundaries between minority and majority class samples. It then applies the SMOTE algorithm to synthesize a larger number of new minority class samples closer to the boundaries, thereby improving the decision-making ability of the classification model in the boundary region. Additional technical information for SVMSMOTE can be found in the literature (Nguyen et al., 2011). In this study, the Python extension pack imbalanced-learn was used to implement this technique.

**Table 3:** Number of epochs.

**Datasets**	**Error events**	**Correct events**	**Total**
BNCI moving cursor	1,322	5,115	6,437
LSC speller	713	3,087	3,800
Gaze speller	511	4,420	4,931
Total	2,546	12,622	15,168

The stratified-Kfold cross-validation (Kohavi et al., 1995) was used to train and validate our pre-trained model because of the restricted quantity of data available. This method can maintain the distribution of the training and validation datasets consistent with the original dataset after splitting. The *K*-value was set to 5. To prevent leakage of the validation set into the training set, oversampling was done at each cross-validation iteration rather than uniformly after the preprocessed data were loaded.

Stochastic gradient descent (SGD; Ruder, 2016) was the optimizer used in the pre-training phase, and the momentum was set to 0.9. The cosine learning rate decay (Loshchilov and Hutter, 2016) was utilized to dynamically lower the learning rate, which was initialized at 0.0001. Since the goal of this study is classification, the loss of model training was calculated using the cross-entropy function. In addition, to reduce the impact of label errors, the label smoothing (Müller et al., 2019) technique was used. The batch size and epochs of the model pretraining were set to 32 and 200, respectively. The entire process was conducted on an NVIDIA GeForce RTX 3080 with 10 GB of RAM, utilizing the Pytorch (Paszke et al., 2019) framework for code implementation.

*Fine-tuning*: Six fine-tuned models would be obtained by using the data from each participant in the BNCI moving cursor dataset to fine-tune the model that performed best in the stratified-Kfold cross-validation during the pre-training stage.

In the fine-tuning phase, the Adam optimizer (Kingma and Ba, 2014) was adopted. Ten times smaller than the pre-training stage, 0.00001, was the starting learning rate. Similarly, cosine rate decay (Loshchilov and Hutter, 2016) was used to dynamically reduce the learning rate. Furthermore, this phase's training epoch was set at 100. The training tools and other parameters are the same as they were during the pre-training stage. Each participant's data was used to independently test each fine-tuned model. For each fine-tuned model, the participant data involved in the fine-tuning process is not involved in the test.

### 2.5 Experiments

Before executing the proposed training strategy, we first performed leave-one-subject-out classification and one-train-one-test classification on the target task, the BNCI moving cursor. The number of epochs per participant in the BNCI moving cursor dataset is shown in Table 4.

**Table 4:** The number of epochs per participant in the BNCI moving cursor dataset.

**Participant**	**1**	**2**	**3**	**4**	**5**	**6**	**Total**
Error events	235	242	188	211	241	205	1,322
Correct events	809	838	848	841	882	897	5,115
Total	1,044	1,080	1,036	1,052	1,123	1,102	6,437

Leave-one-subject-out classification is a pattern that does not rely on the prior knowledge of a new individual. During the same experimental session, data from one participant was chosen at a time to serve as the test set, while the remaining participants' data served as the training set. Afterward, stratified 5-fold cross-validation was used to randomly divide the training and validation sets from the training set. The remaining parameters were consistent with the pre-training phase of the proposed training strategy. The purpose of leave-one-subject-out classification is to verify the feature extraction ability of the proposed model on the target task so as to facilitate a wider comparison with the existing methods.

One-train-one-test classification refers to a pattern in which a randomly initialized model is trained on the data of one participant in the target task and then tested separately on the data of the remaining participants. All parameters of the model training are consistent with the fine-tuning phase of the proposed training strategy. The results obtained from this mode can be used to evaluate the performance that the proposed model can achieve when it is trained without the help of pre-training, providing a performance benchmark for experiments using the complete training strategy. If the proposed model training strategy achieves better results than this classification mode, then the transfer learning-based training strategy is effective.

After completing the above two experiments, the proposed training strategy was executed.

### 2.6 Performance metric

The performance metric is a quantitative indicator of the strengths and weaknesses of the model. Due to the main goal of this study being to improve the accuracy of ErrPs classification in the background of a small dataset, accuracy and area under curve (AUC) were selected as performance metrics.

## 3 Results

For the leave-one-subject-out classification on the BNCI moving cursor task, average accuracy rates of 73.28 and 70.34% were obtained in sessions 1 and 2, respectively. The performance of all six participants is shown in Table 5. The results recorded in the table are the classification accuracy of each participant using the best model of 5-fold cross-validation. For session 1, the classification accuracies of all participants have surpassed 65%, four of six participants have >70% accuracy, and two participants have accuracy over 79%. For session 2, the highest classification accuracy obtained was 86.49%, and the lowest classification accuracy was 60.78%.

**Table 5:** The accuracy of the leave-one-subject-out classification on the BNCI moving cursor task (%).

**Participant**	**1**	**2**	**3**	**4**	**5**	**6**	**Average**
**Session 1**	**78.32**	**66.60**	**70.68**	**79.51**	**79.12**	**65.47**	**73.28**
Session 2	86.49	65.33	60.78	66.91	72.40	70.15	70.34

Table 6 shows the results of the one-train-one-test classification on the BNCI moving cursor dataset. The first row of the table specifies each training set, and the first column specifies each test set. Each column of the table represents the results of testing the remaining participants separately with the optimal model that was selected by 5-fold cross-validation using the training set specified in the column. Each row represents the results of the test on each model by a given participant. Of the six models, five had an average testing accuracy of >74.9%, and three of them exceeded 77%. Overall, for the one-train-one-test classification on the BNCI moving cursor task, a total average accuracy of 75.84% was achieved, which is the average of all one-train-one-test classifications.

**Table 6:** The results of one-train-one-test classification on the BNCI moving cursor task (%).

**Training set Testing set**	**1**	**2**	**3**	**4**	**5**	**6**
1	-	78.93	81.13	73.47	79.02	72.03
2	73.33	-	78.24	76.57	75.83	70.19
3	73.26	79.73	-	80.41	81.76	72.97
4	75.48	78.14	79.94	-	76.52	68.44
5	79.34	78.18	80.41	76.40	-	70.35
6	73.14	76.59	72.41	71.05	72.05	-
Average	74.91	78.31	78.43	75.58	77.04	70.80

Table 7 presents the outcomes of cross-task classification, wherein the model was pre-trained on the LSC speller dataset and subsequently fine-tuned on the BNCI moving cursor dataset. The first row of the table denotes each fine-tuned dataset, while the first column specifies each testing set. Each column in the table represents the results of testing the remaining participants individually using the model fine-tuned from the optimal pre-trained model selected through 5-fold cross-validation. Similar to Table 6, each row represents the test outcomes of each model by a specific participant. As observed in the table, all six models achieved an average testing accuracy exceeding 76%, with four of them surpassing 78%. The overall average accuracy of 78.45% represents the average performance across all classifications in this context. The last row of Table 7 presents the average results of the six models obtained using the baseline classification pattern, one-train-one-test classification. It is evident that the average test accuracy of each model, achieved by fine-tuning the pre-trained model on the target dataset, outperforms that of the one-train-one-test classification. Notably, when participant 6's data was utilized for model training, the corresponding model exhibited the highest increase in average accuracy, with a significant improvement of 6.09%. Overall, the model's total average classification accuracy after the pre-training stage was 2.61% higher compared to the baseline classification.

**Table 7:** The results of cross-task classification which pre-trained on the LSC speller task and fine-tuned on the BNCI moving cursor task (%).

**Fine-tuned set Testing set**	**1**	**2**	**3**	**4**	**5**	**6**
1	-	80.36	81.80	77.01	81.51	77.30
2	75.00	-	77.04	79.63	78.33	74.44
3	76.74	81.95	-	81.95	80.69	79.05
4	75.57	80.13	80.13	-	77.57	77.19
5	80.32	80.68	79.88	78.27	-	76.49
6	76.32	78.31	77.68	77.40	74.86	-
Average	76.79	80.29	79.31	78.85	78.59	76.89
Non-pretrained	74.91	78.31	78.43	75.58	77.04	70.80

Table 8 displays the outcomes of cross-task classification, where the model was pre-trained on the Gaze speller dataset and fine-tuned on the BNCI moving cursor dataset. Similar to Table 7, each column in the table represents the test results for each participant using the model fine-tuned from the optimal pre-trained model selected through 5-fold cross-validation. Each row represents the test outcomes for each model by a specific participant. Among the six models, five achieved an average testing accuracy exceeding 79%, with two surpassing 80%. Similar to the cross-task classification pre-trained on the LSC speller task, a total average accuracy of 77.54% was attained in this scenario. The last row of Table 8 also presents the average results of the six models obtained using the baseline classification pattern, one-train-one-test classification. It is clear that, except when participant 6's data was used for model training, the average test accuracy of each model, achieved through fine-tuning the pre-trained model on the target dataset, exceeds that of the baseline classification. When participant 1's data was utilized for model training, the corresponding model exhibited the highest increase in average accuracy, with a significant improvement of 5.29%. Compared to the baseline classification, the overall average classification accuracy of the model after the pre-training phase was 1.7% higher.

**Table 8:** The results of cross-task classification that pre-trained on the Gaze speller task and fine-tuned on the BNCI moving cursor task (%).

**Fine-tuned set Testing set**	**1**	**2**	**3**	**4**	**5**	**6**
1	-	80.17	80.75	77.11	82.18	72.22
2	75.83	-	77.96	78.98	77.50	65.93
3	83.59	80.69	-	81.76	81.95	67.57
4	77.57	79.47	80.23	-	77.47	62.93
5	83.97	80.68	81.66	78.54	-	64.02
6	80.04	79.49	79.22	78.68	77.95	-
Average	80.20	80.10	79.96	79.01	79.41	66.53
Non-pretrained	74.91	78.31	78.43	75.58	77.04	70.80

## 4 Discussion

In order to address the restricted performance of CML and DL approaches resulting from the size of the ErrPs datasets, this study attempts to provide a general approach for ErrPs classification. To achieve this goal, a DL model combining transformer encoders and convolutional layers was proposed to extract and classify ErrPs features. Furthermore, to train the proposed deep neural network model, a training strategy based on transfer learning's fine-tuning technology was provided. Three publicly accessible ErrPs datasets from various EEG tasks are employed to assess the suggested approach.

In the Results section, we first demonstrate the accuracies of leave-one-subject-out classification on the BNCI moving cursor dataset. To highlight the feature extraction capability of the proposed model, we investigated other methods for ErrPs classification on the BNCI moving cursor dataset. In the leave-one-subject-out classification, Kumar et al. (2018) used the LDA algorithm to classify error events and correct events. Parashiva and Vinod (2020) employed a two-stage trained ANN algorithm to classify ErrPs.

Since most ErrPs datasets contain two sessions of data per participant, many of the existing ErrPs classification methods are specific to within-session classification in a single task or cross-session classification in a single task. Within-session classification in a single task means that model training, model validation, and model testing are all executed on the same session in a single task dataset. For instance, if the dataset includes two sessions, the whole train-test pipeline will execute on sessions 1 and 2, respectively. For cross-session classification in a single task, the specific step is to choose one session data of each participant to be the training set and another session data to be the testing set.

For the within-session classification scenario on the BNCI moving cursor dataset, Torres et al. (2018) designed two CNN-based DL models to classify ErrPs. One of the models only selects data from FCz and Cz channels as input, while another model uses data from all channels as input to the model. Parashiva and Vinod (2021) proposed two electrode ranking methods, the cosine similarity measure and the euclidian distance measure, combined with the LDA algorithm for ErrPs classification. For the cross-session classification scenario on the BNCI moving cursor dataset, Chavarriaga and Millan (2010) used a Gaussian classifier to classify ErrPs. Kumar et al. (2018) used the LDA algorithm to classify ErrPs.

Based on the above investigation, we implemented the classification models used in these studies and conducted experiments in three different classification scenarios for comprehensive comparison. In order to ensure that the dimensions of the input data are unified, for the CNN-based model proposed by Torres et al. (2018), only the model that selects two channel data as input was reproduced.

Table 9 shows the comparison of the proposed method with existing methods on the BNCI moving cursor dataset in the leave-one-subject-out classification scenario. The table lists the average detection rate of erroneous events, the average detection rate of correct events, the average classification accuracy, and the average AUC value for a more thorough comparison. As can be seen, compared to the current methods, for session 1, the model proposed in this paper improved the average classification accuracy by 1.53–10.24% and the average AUC value by 4.36–9.83%. For session 2, the proposed model increased the average classification accuracy by 1.71–13.35% and the average AUC value by 1.26–9.28%.

**Table 9:** The results of leave-one-subject-out classification using different methods (%).

**Method**	**Session 1**				**Session 2**			
	**Error rate**	**Correct rate**	**Accuracy**	**AUC**	**Error rate**	**Correct rate**	**Accuracy**	**AUC**
LDA	56.36	66.78	64.34	66.23	62.64	63.58	63.51	68.83
Gaussian	52.91	77.21	71.75	70.41	70.69	53.58	56.99	68.71
ANN	56.70	65.08	63.04	65.64	52.47	68.21	65.11	64.43
ConvNet: FCz, Cz	54.17	74.59	69.94	71.11	60.15	70.93	68.63	72.45
Proposed method	56.91	77.84	73.28	75.47	64.56	71.41	70.34	73.71

Table 10 shows the comparison of within-session classification using different methods on the BNCI moving cursor dataset. It is obvious that in this classification scenario, the performance of the ANN algorithm in the two sessions is very poor compared to other algorithms. The average classification accuracy in the two sessions is only 60.51 and 64.22%, respectively, and the average AUC value is 63.86 and 65.47%. The CNN-based model performed better on both sessions. Average classification accuracy rates of 78.82 and 78.13% and average AUC values of 82.13 and 84% were achieved, respectively. The model proposed in this paper is slightly better than the comparison methods, with average classification accuracy of 79.17 and 78.31% and average AUC values of 82.94 and 84.23% obtained on sessions 1 and 2, respectively.

**Table 10:** The results of within-session classification using different methods (%).

**Method**	**Session 1**				**Session 2**			
	**Error rate**	**Correct rate**	**Accuracy**	**AUC**	**Error rate**	**Correct rate**	**Accuracy**	**AUC**
LDA	65.88	74.88	72.95	76.91	68.23	73.39	72.33	79.49
Gaussian	64.62	80.06	76.82	79.99	66.77	79.43	77.06	81.39
ANN	54.99	62.00	60.51	63.86	53.33	66.70	64.22	65.47
ConvNet: FCz, Cz	66.37	82.18	78.82	82.13	68.64	80.43	78.13	84.00
Proposed method	69.35	81.82	79.17	82.94	70.09	80.17	78.31	84.23

The results of cross-session classification of the BNCI moving cursor dataset using different methods are shown in Table 11. The Session 1 part of the table represents the results obtained by training the model using data from session 2 and testing it on data from session 1, and vice versa. Similar to within-session classification, the ANN algorithm performs poorly in this classification scenario. For session 1, the proposed model has a minimum improvement of 2.06% average classification accuracy and 4.41% average AUC value compared with the existing methods. For session 2, the proposed model improved the average classification accuracy by a minimum of 0.33% and the average AUC value by at least 2.99%.

**Table 11:** The results of cross-session classification using different methods (%).

**Method**	**Session 1**				**Session 2**			
	**Error rate**	**Correct rate**	**Accuracy**	**AUC**	**Error rate**	**Correct rate**	**Accuracy**	**AUC**
LDA	61.74	74.56	72.03	74.30	62.70	72.06	70.06	73.07
Gaussian	57.30	80.13	75.73	75.67	62.88	79.36	75.90	77.10
ANN	53.55	58.62	57.64	58.71	50.63	67.45	64.03	64.27
ConvNet: FCz, Cz	57.08	79.51	74.96	74.74	61.86	77.79	74.52	76.35
Proposed method	62.18	81.87	77.79	80.08	63.38	79.71	76.23	80.09

Based on the above comparisons, there is no doubt that the proposed model shows better feature extraction ability for ErrPs. However, the experiments discussed above and the comparative experiments were performed only on a single ErrPs dataset. In practical application scenarios, it is challenging to obtain enough training data at one time due to the complexity of ErrPs acquisition processes and the restriction of access rights to ErrPs datasets. Therefore, it is important to use only a small number of samples of the target task to participate in model training to detect the new data generated by new participants in the target task, which is more in line with the needs of practical application.

One-train-one-test classification and leave-one-subject-out classification are both classification patterns that do not rely on prior knowledge of new participants. The test results obtained from new participants can effectively reflect the model's generalization ability across participants. The crucial distinction between these two classification modes is that the leave-one-subject-out classification uses five of six participants to participate in the model training stage, leaving only one participant as the test set, while the one-train-one-test classification uses only one participant's data as the training set and then tests each of the remaining participants separately. The one-train-one-test classification could significantly reduce the number of target EEG task samples used in model training, which aligns well with scenarios where there is a scarcity of training data specific to the target task, as often encountered in practical applications. In order to improve the classification performance of the model in this scenario, we carried out the exploration of cross-task, cross-subject classification using the proposed training strategy. The core idea is to use the existing ErrPs dataset to assist in the classification of ErrPs in the new EEG task. The cross-task classification results presented in Tables 7, 8 validate the effectiveness of the proposed training strategy.

**Figure 2:**
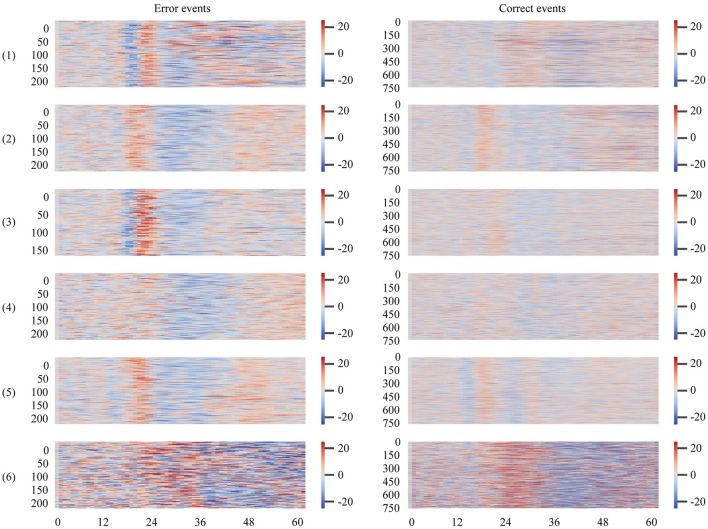
ErrPs plots of all participants in the BNCI moving cursor dataset. The time window spans the period from the start of the event to its end. The value on the horizontal axis represents the number of sampling points of the extracted epoch, and the vertical axis represents each epoch, arranged from top to bottom according to the chronological order of the events.

It can be noted that the overall test performance of participant 6 in these classification modes was significantly lower than that of the rest of the participants. To explore the reasons, we plotted the ErrPs pictures of all participants in the BNCI moving cursor dataset, shown in Figure 2. The closer the color in the graph is to dark blue, the more negative the amplitude value of the event at the current sampling point, and the greater the absolute value. Correspondingly, the event's amplitude value at the present sampling point is larger the closer the color is to dark red.

As shown in Figure 2, the amplitude values of error events for participants 1–5 in the BNCI moving cursor dataset show approximately the same trend. This trend refers to the significant negative deflection of the waveforms of all erroneous events over a highly coincident time frame (in Figure 2, where the high positive amplitude shifts to the low negative amplitude). This negative wave is followed by a significant positive deflection that changes from negative (blue) to positive (red). When it comes to the occurrence of positive and negative deflection, the timing differences between those participants are relatively insignificant. However, the ErrPs plot of the 6th participant was significantly different from that of the first five participants. Similar to the error events, we can observe that the amplitude variation range and trend of the correct events for participants 2–5 in the BNCI moving cursor dataset are close. However, the first participant's is slightly different from them, and the sixth participant's is significantly different, with a wider range of amplitude values.

The above information indicates that the waveform, amplitude, crest, and other main characteristics of the error event and the correct event of the 6th participant are quite different from the rest of the participants. This may be the result of low signal quality from improper experimental operation or the poor physiological state of the individual during the EEG experiment. This is probably the main reason why the model did not perform well with the sixth participant.

## 5 Conclusion

In this investigation, a new approach based on DL and transfer learning for classifying ErrPs was proposed. The method consists of a feature extraction network combining convolutional layers and transformer encoders and a model training strategy based on fine-tuning technology. The proposed method was evaluated on three publicly available datasets. Among them, the LSC Speller and Gaze Scaler datasets are used as the source task datasets, and the BNCI moving cursor dataset is used as the target task dataset. The results exceeded our baseline, achieving an average accuracy of about 78%. In addition, the performance of the proposed model in the leave-one-subject-out, within-session, and cross-session classification scenarios significantly exceeds that of the existing methods on the target task dataset, reaching an average accuracy of 71.81, 78.74, and 77.01%, respectively. These outcomes demonstrate the effectiveness of the proposed model in capturing the electrode and time features of ErrPs. Simultaneously, they also reflect the efficacy of the proposed training strategy in utilizing existing ErrPs datasets to assist in ErrPs classification for new EEG tasks. This aligns with our initial intention of trying to carry out cross-task knowledge transfer for ErrPs, mitigating the issue of limited datasets in the ErrPs field to a certain extent. Overall, this study provides a fresh idea for deep learning techniques' performance bottleneck stemming from the scale constraints of ErrPs datasets, and it may serve as a source of inspiration for other studies focused on ErrPs and its uses.

## Data availability statement

Publicly available datasets were analyzed in this study. This data can be found at: BNCI cursor moving: https://bnci-horizon-2020.eu/database/data-sets; LSC speller: https://dx.doi.org/10.21227/6wpz-g759; Gaze speller: https://doi.org/10.6084/m9.figshare.5938714.v1.

## Author contributions

GR: Conceptualization, Investigation, Methodology, Software, Writing—original draft, Writing—review & editing. AK: Conceptualization, Investigation, Writing—review & editing. SM: Writing—review & editing, Conceptualization. QF: Conceptualization, Funding acquisition, Supervision, Writing—review & editing.
